# Effects of Training Health Workers in Integrated Management of Childhood Illness on Quality of Care for Under-5 Children in Primary Healthcare Facilities in Afghanistan

**DOI:** 10.15171/ijhpm.2019.69

**Published:** 2019-09-03

**Authors:** Essa Tawfiq, Sayed Ali Shah Alawi, Kayhan Natiq

**Affiliations:** ^1^Department of Epidemiology and Biostatistics, School of Population Health, The University of Auckland, Auckland, New Zealand.; ^2^Child and Adolescent Health Department, Afghan Ministry of Public Health, Kabul, Afghanistan.; ^3^Silk Route Training and Research Organization (SRTRO), Kabul, Afghanistan.

**Keywords:** IMCI, Training, Quality of Care, Primary Healthcare, Afghanistan

## Abstract

**Background:** Training courses in integrated management of childhood illness (IMCI) have been conducted for health workers for nearly one and half decades in Afghanistan. The objective of the training courses is to improve quality of care in terms of health workers communication skills and clinical performance when they provide health services for under-5 children in public healthcare facilities. This paper presents our findings on the effects of IMCI training courses on quality of care in public primary healthcare facilities in Afghanistan.

**Methods:** We used a cross-sectional post-intervention design with regression-adjusted difference-in-differences (DiD) analysis, and included 2 groups of health workers (treatment and control). The treatment group were those who have received training in IMCI recently (in the last 12 months), and the control group were those who have never received training in IMCI. The assessment method was direct observation of health workers during patient-provider interaction. We used data, collected over a period of 3 years (2015–2017) from primary healthcare facilities, and investigated training effects on quality of care. The outcome variables were 4 indices of quality care related to history taking, information sharing, counseling/medical advice, and physical examination. Each index was formed as a composite score, composed of several inter-related tasks of quality of care carried out by health workers during patient-provider interaction for under-5 children.

**Results:** Data were collected from 733 primary healthcare facilities with 5818 patients. Quality of care was assessed at the level of patient-provider interaction. Findings from the regression-adjusted DiD multivariate analysis showed significant effects of IMCI training on 2 indices of quality care in 2016, and on 4 indices of quality care in 2017. In 2016 two indices of quality care showed improvement. There was an increase of 8.1% in counseling/medical advice index, and 8.7% in physical examination index. In 2017, there was an increase of 5.7% in history taking index, 8.0% in information sharing index, 10.9% in counseling/medical advice index, and 17.2% in physical examination index.

**Conclusion:** Conducting regular IMCI training courses for health workers can improve quality of care for under-5 children in primary healthcare facilities in Afghanistan. Findings from our study have the potential to influence policy and strategic decisions on IMCI programs in developing countries.

## Background

Afghanistan has achieved significant improvement in reducing under-5, infant and neonatal mortality since 1990. Despite that, child mortality and morbidity in Afghanistan remains among the highest in the world. According to the Afghanistan Demographic Health Survey 2015, the neonatal mortality rate was 22 deaths per 1000 live births, the infant mortality rate was 45 deaths per 1000 live births, and the under-5 mortality rate was 55 deaths per 1000 live births, meaning that one out of every 18 children died before reaching their fifth birthday.^1^ Four-fifths of all deaths in the first 5 years of life occurred during infancy. Data prior to 2010 shows even worse child mortality in the country.^2^ The Afghanistan Demographic Health Survey 2015 indicates that child mortality stills remains unacceptably high.

Five primary causes of child mortality are pneumonia, diarrhea, measles, malaria, and malnutrition, and they account for over 70% of child death.^3^ These 5 diseases are responsible for more than half of the estimated 12.7 million deaths annually in children aged under-5 years.^4^ To reduce death and illnesses related to these diseases, the World Health Organization (WHO) and the United Nations Children’s Fund (UNICEF) jointly initiated and developed the Integrated Management of Childhood Illnesses (IMCIs) in early 1990s.^5,6^ IMCI aims to significantly reduce the morbidity and mortality among children under-5 years old, and to contribute to their healthy growth and development through increasing clinical knowledge and skills of health workers, and encouraging active involvement of families and communities in healthcare seeking behavior for children.

In a health facility, IMCI begins with a health worker inquiring about danger signs and symptoms in a child with any sign and symptom suggestive of acute respiratory infection (ARI), diarrhea, malaria, measles, malnutrition, and anemia. The presence of danger signs and symptoms indicates urgent care and treatment, and the child either to be admitted in the facility or referred to a higher level facility in accordance with IMCI guidelines. When the child does not show danger signs and symptoms, the health worker assesses and classifies the child for treatment plan, medical advice and follow-up visits. The health worker discusses and counsels with the child’s caregiver about home care, immunization, nutrition, breast-feeding, treatment plan, and danger signs and symptoms. In several African countries, it has been found that after health workers received IMCI training, they followed IMCI guidelines more extensively when assessing and treating unwell children and when providing medical advice to the child’s caregiver.^4,5,7,8^ There is little research available on the subject in Afghanistan. Our study investigated effects of IMCI training courses on health workers performance in terms of history taking, information sharing, counseling/medical advice, and physical examination for under-5 children in primary healthcare facilities in Afghanistan.

In Afghanistan, health workers receive IMCI training according to the national IMCI training guidelines. The training courses are conducted by the IMCI department in close coordination with the implementing partners who are non-governmental organizations (NGOs) and who manage public healthcare facilities on behalf of the Afghan Ministry of Public Health (MoPH). The IMCI department is one of the MoPH’s departments, and the department has master trainers. By background, the master trainers are medical doctors who have received comprehensive training to become master trainers. IMCI training courses are conducted at regional/provincial training centers, including practical sessions that are conducted in selected health facilities at the capital of region/province. Each health facility is required to provide the IMCI guidelines to the health workers. The health workers follow the standard protocols from the guidelines for assessing the unwell children, for making classification of and managing the children’s illnesses, and for providing counseling and medical advice to the children’s caregivers. In this study, we hypothesized that conducting IMCI training for health workers can improve their performance in terms of history taking, information sharing, counseling/medical advice, and physical examination for under-5 children.

## Methods

### Study Design, Sampling and Data Sources

This is a cross-sectional post-intervention study with a regression-adjusted difference-in-differences (DiD) component. There were 2 groups of health workers: treatment and control groups. The treatment group were health workers who have received IMCI training recently (within the past 12 months), and the control group were health workers who have never received IMCI training. This study is part of the Annual Health Facility Performance Assessment (AHFPA) conducted in 2015, 2016, and 2017. The AHFPA was conducted in 733 randomly selected primary healthcare facilities, composed of 3 types of facility that included 219 sub-health centers (SHCs), 356 basic health centers (BHCs), and 158 comprehensive health centers (CHCs). At each round of AHFPA, primary healthcare facilities were stratified by facility type. The required quota on each type was randomly selected for each of the 34 provinces of Afghanistan. Different health facilities were selected randomly at each round of AHFPA; however, it is possible that same health facilities may have been selected randomly in the consecutive rounds of AHFPA. In this study we call these facilities as resampled health facilities. In each healthcare facility, up to ten patients were selected through systematic random sampling of patients. After obtaining the consent of the child’s caregiver, the patient-provider interaction was observed by the assigned surveyor. By background, the surveyors were medical staff who had received extensive training on how to conduct the AHFPA survey. During patient-provider interaction, each surveyor recorded responses, using the survey questionnaire containing questions related to the tasks on quality of care that a health worker is required to exert when providing IMCI services for under-5 children. Then the health worker was asked as whether he/she had received IMCI training in the past. Three response options were read to the health worker, and his/her responses were recorded in the questionnaire. The 3 response options were: ‘Yes, I have received training in IMCI within the last 12 months,’ ‘Yes, I have received training in IMCI but not within the last 12 months,’ ‘No, I have never received training in IMCI.’ Demographic information of patients and health workers was also collected. For data collection 2 survey questionnaires were used: one for children under-5 and another for patients 5 years and older. For the purposes of this study, we analyzed the data collected from children under-5 years of age who were brought to the healthcare facilities due to having any of the 5 complaints of fever, diarrhea, cough, sore throat, and vomiting. These complaints are mainly related to IMCI illnesses in children. We set 3 inclusion criteria for the study. First, a child with at least one of the 5 complaints was eligible for inclusion. Second, duration of the complaint(s) must be up to 14 days prior to the visit. Third, health workers who have received IMCI training recently (in the last 12 months) were included. However, health workers who have received IMCI training beyond the last 12 months were excluded. This is because health workers reporting training beyond 12 months could have been part of the recent training group of health workers in the resampled health facilities in 2016 and 2017, and this could have biased the IMCI training effects if they were included in the study. The control group were those health workers who have never received training in IMCI.

### Selection of Health Workers for IMCI Training Courses

In Afghanistan IMCI is one of the components of Basic Package for Health Services,^9^ and each health worker managing under-5 children in public healthcare facilities is required to follow the IMCI algorithm. Health workers from public healthcare facilities are selected for IMCI training courses after their training needs have been assessed by their managers (eg, the health facility in-charge, the NGO manager or local health authority). Each round of a training course lasts for 2 weeks. After completing the course, the health workers who attended the course go back to their respective healthcare facilities where they receive regular supervision and on-the-job training, including IMCI, by the NGO responsible to manage the healthcare facilities.

### Selection of Tasks and Development of Quality Care Indices

The tasks selected for developing indices to assess quality of care correspond to those recommended by the IMCI guidelines developed in Afghanistan. Quality of care was assessed through direct observation at the level of patient-provider interaction. It is expected that health workers working in public healthcare facilities comply with the guidelines by exerting the tasks when they provide healthcare for under-5 children. The tasks can be categorized into 4 indices – taking history of the child, sharing information with the child’s caregiver, providing counseling and medical advice to the child’s caregiver, and conducting physical examination of the child. When computing the indices, we did not include data on 6 tasks as either the collected information was subject to bias and misinterpretation or it was not relevant to the circumstances of the children being examined. The 6 tasks were: whether the health worker (1) checks if the child has a change in the level of consciousness, (2) asks about convulsions, (3) asks if the child has passed measles, (4) looks at the child’s both feet and ankles for edema, (5) examines some parts of the child’s body either by close inspection or actual contact, and (6) prescribes medicine for the child. Data on the above questions were collected from all children for whom the health workers provided IMCI services. However, when relevant question was asked for the children who had a specific condition, the denominator was defined as the number of children with the specific condition. For example, the denominator for duration of fever was defined as number of children who had fever.

Table 1 shows the 4 quality care indices and related tasks. The indices are history taking, information sharing, counseling and advice, and physical examination. There are 13 tasks in the history taking index, and the tasks are: whether the health worker (1) greets the child or his/her caregiver, (2) asks the child’s age, (3) asks about the child’s complaints and the duration, (4) asks if the child has fever, (5) asks the duration of fever if the child has fever, (6) asks if the child can drink or suckle breast milk, (7) asks if the child vomits, (8) asks if the child has diarrhea, (9) asks the duration of diarrhea if the child has diarrhea, (10) ask if the child has bloody diarrhea in a child with diarrhea, (11) asks if the child has a cough, (12) asks the duration of cough for a child with cough, and (13) asks if the child has received any treatment for the present illness. There are 6 tasks in the information-sharing index, and the tasks are: whether the health worker (1) tells the child’s caregiver the name of the illness the child is suffering from, (2) describes the course of the illness to the child’s caregiver, (3) tells the child’s caregiver the name of the drug(s) the health worker has prescribed, (4) explains about possible side effects of the prescribed drug(s), (5) describes how the child’s caregiver should give the prescribed drug(s) to the child, and (6) describes how the child’s caregiver look after the child at home because of the illness. There are 10 tasks in the counseling and medical advice index, and the tasks are: whether the health worker (1) discusses about signs and symptoms that prompt actions of the child’s caregiver to take the child to a health facility, (2) discusses with the child’s caregiver about follow-up visits for the child, (3) checks if the child is due for vaccination, (4) sends the child for vaccination if the child was due for vaccination, (5) checks if the child is due for receiving vitamin A, (6) provides vitamin A to the child if the child was due for it, (7) discusses with caregivers of children under-2 years about the importance of breastfeeding, (8) discusses with the child’s caregiver about feeding children during illnesses, (9) discusses with the child’s caregiver about supplementary feeding for young children, and (10) asks if the child’s caregiver has any question. There are nine tasks in the physical examination index, and the tasks are: whether the health worker (1) weighs the child, (2) measures the child’s height, (3) measures by tape the child’s mid-upper arm circumference, (4) examines the child for anemia (eg, looking at the child’s palms, lower eyelids, and nails), (5) uses a thermometer to check if the child has fever, (6) counts respiratory rate if the child has a cough, (7) listens to the child’s chest by statoscope if the child has a cough, (8) examines skin turgor if the child has diarrhea (eg, testing elasticity of the child’s any arm’s or leg’s skin), and (9) examines the child’s anterior fontanel if the child has diarrhea and the child is under-9 months of age.

**Table 1:** List of the 4 Quality Care Indices and Related Tasks That Health Workers Carried out During Patient-Provider Interaction for Under-5 Children

**Index**	**Task**
History taking	Health worker greets the child’s caregiver/patient
	Health worker asks the child’s age
	Health worker asks about the child’s complaint(s) and the duration
	Health worker asks whether the child has fever
	Health worker asks about the duration of fever if the child has fever
	Health worker asks whether patient can drink or suckle breast milk
	Health worker asks whether the child vomits
	Health worker asks whether the child has diarrhoea
	Health worker asks about the duration of diarrhoea if the child has diarrhoea
	Health worker asks about bloody diarrhoea if the child has diarrhoea
	Health worker asks if the child has a cough
	Health worker asks about the duration of the cough if the child has a cough
	Health worker asks whether the child has received any treatment for the illness
Information sharing	Health worker tells the child’s caregiver the name of illness the child is suffering from
	Health worker describes the course of illness
	Health worker tells the child’s caregiver the name of drug(s) described
	Health worker explains about possible side effects of the drug(s) prescribed
	Health worker describes to the caregiver how to give the prescribed medicine to the child
	Health worker describes to the child’s caregiver about home care for the child
Counselling and advice	Health worker describes signs and symptoms that prompt urgent actions
	Health worker describes to the child’s caregiver about follow-up visits
	Health worker checks whether the child is due for vaccination
	Health worker sends the child for vaccination if vaccination was due
	Health worker asks whether the child is due to receive vitamin A
	Health worker provides vitamin A for the child if he/she was due for vitamin A
	Health worker discusses about breastfeeding for under-2 children
	Health worker discusses about feeding children during the illness
	Health worker discusses about supplementary feeding for the child
	Health worker asks whether the child’s caregiver has any question
Physical examination	Health worker weighs the child
	Health worker measures the child’s height
	Health worker measures the child’s mid-upper arm circumference
	Health worker examines the child for anaemia
	Health worker checks with thermometer if the child has fever
	Health worker counts Respiratory Rate in patients who has a cough
	Health worker listens to the child’s chest by statoscope if the child has a cough
	Health worker examines the child’s skin turgor if the child has diarrhoea
	Health worker examines anterior fontanel in under-9 months old child with diarrhoea

Outcome variables were defined as the 4 indices. Each index was computed as a composite score of the related tasks. The proportion of tasks carried out by a health worker out of total tasks the health worker was supposed to perform for the index was computed on scale of 0.0 to 1.0.

Confounding variables of IMCI training were defined as types of health facilities, types of health workers, and the year of program implementation. Variables such as patient demography and socioeconomic status were not considered as confounders of IMCI training of health workers, because these variables are less likely to affect health workers performance during patient-provider interaction for under-5 children. However, we defined patient socioeconomic status for descriptive analysis. Socioeconomic status was defined based on the ownership of household assets and state of household amenities reported by the children’s caregivers. Using the ownership of household assets and amenities has been found to be a valid and pragmatic approach to assess patient socioeconomic status in developing countries.^10,11^ For descriptive analysis in our study, we used ownership of 13 household assets (eg, refrigerator) and 8 household amenities (eg, main material used for roofing). Data on the ownership of household assets was collected on binary responses (owned = 1, and did not own = 0), and for household amenities, the data collected was recoded as 1 if the amenity reported was at an acceptable category (eg, if metal, wood, bricks, concrete, or tiles were used as the main material for roofing, then it was recorded as (1) otherwise it was recoded as zero).

### Statistical Model

A DiD component was incorporated into the study design to measure IMCI training effects on quality of care over 1-year (2016) and over 2-year time (2017). Quality of care was defined as the score of quality care for history taking, information sharing, counseling/medical advice, and physical examination for under-5 children. A multivariate regression model with DiD component was utilized. The model has been modified from a recently published paper,^12^ and is shown below.

$$\begin{array}{l} Y_{ijt}\;=\;\beta_{0}+\beta_{1}IMCI_{jt}+\beta_{2}yr2016_{jt}+\beta_{3}yr2017_{jt}+\beta_{4}IMCI_{jt}\;\\ {}*\;yr2016+\beta_{5}IMCI_{jt}*yr2017+\sum_{K=1}^{K}\beta_{j}X_{ijt}\text{+}\varepsilon_{ijt}\; \end{array}$$

*Y*_ijt_ refers to the score of quality care for health worker *i* in health facility *j* at time *t. IMCI* is the dummy variable for IMCI training (within the last 12 months); and*yr2016*_jt_*, yr2017*_jt_ are dummy variables for year 2016 and 2017 (2015 is the reference year). *IMCI* * yr2016 and IMCI* yr2017 are interaction terms between IMCI training and years 2016 and 2017. *X*_ijt_ denotes the vector of confounders, and *k* refers to the number of confounders. *β*_0_ stands for the intercept which is the score of quality care in 2015 in the control group, and *β*_1_ refers to the difference in the score of quality care between training group and control group in 2015. *β*_2_* and β*_3_ denote changes in the score of quality care in the control group in 2016 and 2017. *β*_4_* and β*_5_ represent IMCI training effects over time in 2016 and 2017.

IMCI training effects or the coefficients obtained by DiD are the differences in the mean scores for quality care between the treatment group and control group. Standard errors for the coefficients were adjusted to account for intra cluster correlation of data collected from repeated samples of health facilities over time.^13^ Failure to adjust variance for the clustering of data can lead to overestimation of treatment effects.^14,15^ In addition to the results obtained from the DiD adjusted multivariate regression, we also examined results using linear regression model. For this, we used cross-sectional pool of data collected over the 3-year time, and compared quality of care between the treatment group and control group, stratified by the confounders of IMCI training. The results identify the difference in mean scores for quality of care between various categories of the confounders. The confounders were included and controlled in the DiD multivariate regression analysis. Statistical significance level was determined at 5% alpha level. All analysis was done, using STATA version 12.^16^

## Results

### Study Characteristics

Table 2 shows patient and provider characteristics in primary healthcare facilities. In total, there were 5818 under-5 children, composed of 2787 in the no training group (control) and 3031 in the training (treatment) group. The mean age of under-5 children was nearly 25 and 27 months in the 2 groups. The proportions of boys and girls were similar in both groups. In terms of mother’s education, the proportions of mothers with no education were highest in both groups (84% vs. 80%); the proportions of mothers with primary education were the same in both groups (6%); and similar proportions (6% vs. 7%) were seen for mothers with intermediate education and for mothers with secondary/higher education in both groups (5% vs. 7%).

**Table 2:** Study Characteristics in Primary Healthcare Facilities (2015–2017)

**Characteristics**	**No Training**	**Training**
**Patients**	**n = 2787**	**n = 3031**
Age (in months)	24.9	26.8
Gender, %		
Boys	51.0	52.0
Girls	49.0	48.0
Mother's education, %		
No education	83.5	80.0
Primary education	5.5	6.0
Intermediate education	6.0	7.0
Secondary/higher education	5.0	7.0
Socioeconomic status, %		
Poorest	11.0	10.0
Poor	51.5	47.0
Not poor	32.0	33.0
Rich	5.0	9.0
Wealthiest	0.5	1.0
**Providers**	**n = 360**	**n = 373**
Type of health worker, %		
Doctor	32.0	44.0
Nurse/doctor assistant	56.0	54.0
Midwife/community midwife	8.0	1.5
Other	4.0	0.5
Gender of health worker, %		
Male	88.0	94.0
Female	12.0	6.0
Type of health facility, %		
Basic health centre	45.0	52.0
Comprehensive health centre	21.0	22.0
Sub health centre	34.0	26.0
Resampled health facilities (2015–2016)	n = 134	n = 149
Resampled health facilities (2016–2017)	n = 85	n = 142

Training refers to IMCI training that health workers reported they had received within last 12 months.

With respect to socioeconomic status, the proportions of patients in the poorest group (0 to 4 out of 21 assets and amenities) were 11% and 10% in the 2 groups of health workers. The majority of patients were from the poor group (5 to 8 out of 21 assets and amenities) and were 52% in no training group and 47% in training group of health workers. The proportions of patients in the not poor group (9 to 12 out of 21 assets and amenities) were 32% and 33% in the 2 groups of health workers. Smaller proportions of patients were from the rich group (13 to 16 out of 21 assets and amenities) with 5% in the no training group and 9% in training group of health workers, and the smallest proportions of patients belonged to the wealthiest group (17 to 21 out of 21 assets and amenities) with 0.5% and 1.0% in 2 groups of health workers. For provider characteristics, there was a smaller proportion of doctors in the no training group than training group (32% vs. 44%); but there were similar proportions of nurses/assistant doctors in the 2 groups (56% vs. 54%). The biggest difference was in the proportions of midwives in the 2 groups (8% vs. 2%). Other types of health workers included pharmacists, lab technicians, and community worker supervisors, and they composed a small proportion of health workers in the 2 groups (4% vs. 0.5%). It is less likely that this category of health workers was involved in making clinical diagnosis and treatment of patients with IMCI related illnesses. With respect to health facilities types, the proportion of BHCs was smaller in the no training group than the training group (45% vs. 52%); but the proportion of SHCs was higher in the no training group than the training group (34% vs. 26%). Similar proportions of CHCs were seen in the 2 groups (21% vs. 22%). The number of resampled facilities is also shown in Table 2.

Figure shows scores of quality care indices between the treatment group and control group in 2015, 2016, and 2017. The scores were higher in the treatment group than the control group across the 4 quality care indices in the 3 years. Noticeable differences were seen in 2016 and 2017 in comparison to 2015 in the indices of information sharing, counseling/advice, and physical examination. For example, the difference between treatment group and control group for physical examination index was over 5% in 2015, over 10% in 2016, and over 20% in 2017. A similar trend was seen for the other 2 indices. However, the difference in the history taking index was smaller than that of the other indices. For example, the difference between treatment group and control group was nearly 2% in 2015, 5% in 2016, and 10% in 2017.

**Figure:**
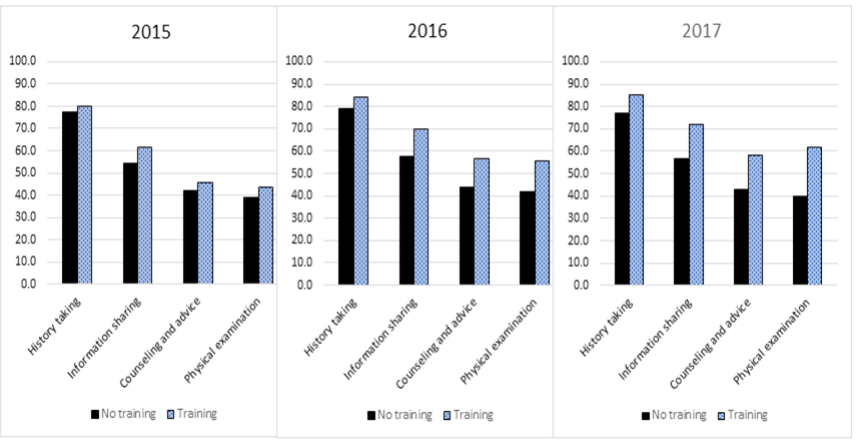
Quality Care Indices by IMCI Training Status of Health Workers. Abbreviation: IMCI, Integrated Management of Childhood Illness.

### Comparison of Mean Scores of Quality Care

Table 3 shows findings from regression analysis across strata of health worker types, health facility types, and the 3 years. Differences in mean scores of quality care are shown between the treatment group and control group for the 4 indices. For example, for doctors the difference was 5% in history taking index, 12% in information sharing index, 11% in counselling/medical advice index, and 14% in physical examination index.

**Table 3:** Quality of Care Indices, Stratified by Type of Health Workers, Type of Health Facilities, and Year of Implementation

**Index**	**Training Status**	**Health Worker**	**Mean Score**	**Difference in Mean Score**	***P*** **Value**	**Facility Type**	**Mean Score**	**Difference in Mean Score**	***P*** **Value**	**Year**	**Mean Score**	**Difference in Mean Score**	***P*** **Value**
History taking	No training	Doctors	0.79	Ref		CHCs	0.78	Ref		2015	0.77	Ref	
	Training		0.84	0.05	.00		0.80	0.02	.21		0.80	0.02	.08
	No training	Nurses	0.77	Ref		BHCs	0.78	Ref		2016	0.79	Ref	
	Training		0.83	0.06	.00		0.84	0.06	.00		0.84	0.05	.00
	No training	Midwives	0.79	Ref		SHCs	0.78	Ref		2017	0.77	Ref	
	Training		0.80	0.01	.82		0.84	0.06	.00		0.85	0.08	.00
Information sharing	No training	Doctors	0.57	Ref		CHCs	0.58	Ref		2015	0.54	Ref	
	Training		0.69	0.12	.00		0.65	0.07	.02		0.62	0.07	.00
	No training	Nurses	0.56	Ref		BHCs	0.56	Ref		2016	0.57	Ref	
	Training		0.68	0.12	.00		0.69	0.14	.00		0.70	0.13	.00
	No training	Midwives	0.59	Ref		SHCs	0.56	Ref		2017	0.57	Ref	
	Training		0.64	0.05	.49		0.68	0.13	.00		0.72	0.15	.00
Counselling/medical advice	No training	Doctors	0.44	Ref		CHCs	0.45	Ref		2015	0.42	Ref	
	Training		0.55	0.11	.00		0.51	0.06	.03		0.46	0.04	.14
	No training	Nurses	0.43	Ref		BHCs	0.44	Ref		2016	0.44	Ref	
	Training		0.54	0.11	.00		0.56	0.12	.00		0.56	0.12	.00
	No training	Midwives	0.44	Ref		SHCs	0.40	Ref		2017	0.43	Ref	
	Training		0.41	-0.04	.61		0.52	0.12	.00		0.58	0.15	.00
Physical examination	No training	Doctors	0.42	Ref		CHCs	0.42	Ref		2015	0.39	Ref	
	Training		0.56	0.14	.00		0.53	0.11	.00		0.43	0.04	.09
	No training	Nurses	0.41	Ref		BHCs	0.42	Ref		2016	0.42	Ref	
	Training		0.53	0.13	.00		0.53	0.12	.00		0.56	0.14	.00
	No training	Midwives	0.37	Ref		SHCs	0.37	Ref		2017	0.40	Ref	
	Training		0.32	-0.05	.45		0.54	0.17	.00		0.62	0.22	.00

Abbreviations: SHCs, Sub-health centers, BHCs, basic health centers, CHCs, comprehensive health centers. Training refers to IMCI training that health workers reported they had received within last 12 months. The difference in mean scores refers to the coefficient obtained by linear regression analysis. The category of nurses also includes assistant doctors.

Among assistant doctors/nurses, a similar trend; though with modest increases in the 4 indices was seen. As for health facility types, in SHCs the difference was 6% in history taking index, 13% in information sharing index, 12% in counseling/medical advice index, and 17% in physical examination index. In BHCs a similar trend to that of SHCs was seen. In CHCs the differences were 2%, 7%, 6%, 11% for the 4 indices, respectively. As for changes in indices over time, the differences in history taking were 2% in 2015, 5% in 2016, and 8% in 2017; for information sharing the differences were 7% in 2015, 13% in 2016, and 15% in 2017; for counseling/medical advice the differences were 4% in 2015, 12% in 2016, and 15% in 2017; and for physical examination the differences were 4% in 2015, 14% in 2016, and 22% in 2017.

### Treatment Effects

Table 4 presents treatment effects obtained by difference in differences multivariate analysis in 2016 and 2017. In 2015, the treatment effect, which corresponds to *β*_1_ as defined in the methods, refers to the difference in mean scores between training group and no training group, adjusted for the confounders. But in 2016 and 2017 treatment effects take account of the changes in quality care over time, and this is what the difference in differences analysis does. The treatment effects in 2016 and 2017 correspond to *β*_4,_ *β*_5_ as defined in the methods. In 2015, the difference in mean scores between treatment group and control group was 2.3% (*P* = .098) for history taking index, 6.7% (*P* = .006) for information sharing index, 3.4% (*P* = .184) for counseling and medical advice index, and 3.6% (*P* = .178) for physical examination index. Over time improvement was seen in 2 indices in 2016, and in all the 4 indices in 2017. For history taking index, in 2016 the training effect was 2.3% (*P* = .195); but it was higher in 2017 at 5.7% (*P* = .006); for information sharing index, in 2016 the effect was 5.6% (*P* = .087); but it was higher in 2017 at 8.0% (*P* = .027); for counseling/medical advice index, in 2016 the training effect was 8.1% (*P* = .018); but it was higher in 2017 at 10.9% (*P* = .003); and for physical examination index, in 2016 the training effect was 8.7% (*P* = .015); but it was higher in 2017 at 17.2% (*P* = .000).

**Table 4:** Treatment Effects of IMCI Training on Quality of Care for Under-5 Children

**Quality Care Index**	**2015**		**2016**		**2017**		**N**
	**Training Effect**	***P*** **Value**	**Training Effect**	***P*** **Value**	**Training Effect**	***P*** **Value**	
History taking	0.023	.098	0.023	.195	0.057	.006	5818
	(0.014)		(0.018)		(0.021)		
Information sharing	0.067	.006	0.056	.087	0.080	.027	5817
	(0.024)		(0.033)		(0.036)		
Counselling/advice	0.034	.184	0.081	.018	0.109	.003	5815
	(0.026)		(0.034)		(0.037)		
Physical examination	0.036	.178	0.087	.015	0.172	.000	5818
	(0.027)		(0.036)		(0.038)		

Abbreviation: IMCI, Integrated Management of Childhood Illness. Training effect, which was obtained by difference in differences multivariate analysis, refers to the difference in mean quality scores between no-training group and training group. Adjusted standard errors are provided in brackets

## Discussion

Findings from our study showed that IMCI training provided to health workers within the last 12 months had positive impact on quality of care for under-5 children. The gains were significant for 2 indices of counseling/medical advice and physical examination in 2016. The other 2 indices showed some improvement in 2016; but the gains were not statistically significant. In 2017, the gains were significant for the 4 indices of history taking, information sharing, counseling/medical advice, and physical examination. A likely reason could be that since nearly two-fifths of the data came from the resampled facilities between 2016 and 2017, it is possible that the gains in 2016 further improved in 2017. This appears to be the case as performance of the facilities resampled were assessed as part of the AHFPA in 2016, and until next round of AHFPA in 2017 the health workers in these facilities had received supervision and on-the-job training by the respective NGO(s) as a requirement of the initial IMCI courses. Some health workers may have perceived that supervisions and on-the-job trainings were part of the initial IMCI courses, even if the courses may have been conducted more than 12 months prior to the day the AHFPA was conducted in the respective health facilities. Also the fact that the difference in quality of care by training changes over time suggests that the same health workers may have been repeatedly trained or that training improved over this time or that other interventions such as supervision and on-the-job training occurred in health facilities. Our findings also suggest that when health workers receive IMCI training regularly, quality of care in terms of history taking, information sharing, counseling/medical advice, and physical examination can improve.

In this study we assessed quality of care through direct observation at the level of patient-provider interaction. The indices used in our study can measure process of care during patient-provider interaction; however, other aspects of quality such as structural quality, validation of diagnoses and managements, patient outcomes, patient perceived quality and satisfaction, and patient health seeking behavior cannot be measured by our study. Research in developing countries shows that structural quality such as availability of infrastructure, equipment, and structural inputs are not associated with higher clinical quality of care in primary healthcare facilities.^17^ Validation of diagnoses and management of IMCI related illnesses refers to the correct diagnosis and management a health worker makes when he or she provides healthcare for a child. This aspect is very important to study; however, a major challenge is the cost of data collection and hiring gold standard surveyors who would correctly diagnose patients and provide treatment plans, and then validate the diagnoses and management plans made by the respective health worker for each patient. Research from a developing country shows positive effects of training health workers on correctly diagnosing and managing IMCI patients.^18^ With respect to patient outcomes, perceived quality and patient satisfaction, and patient health seeking behavior in the context of IMCI training program, we recognize that these aspects are important; but these are not targeted directly by the current IMCI training program. Furthermore, the indices in our study cannot measure quality of care specific to patients suffering from ARI, diarrheal disease, malaria, malnutrition, and measles separately. The reason concerns the purpose of our study as we intended to investigate the effects of IMCI training on quality of care during patient-provider interaction for all children suffering from IMCI related illnesses.

It is expected that findings from our study can have policy implications related to IMCI training programs. The indices can measure compliance of health workers with IMCI recommended guidelines implemented during patient-provider interaction. The index on history taking includes tasks related to building relationships with patients, and identifying reasons that may prompt caregivers to seek care for their children. The index on information sharing includes tasks related to telling patients about their illnesses, the drugs prescribed, and about patient homecare. The index on counseling/medical advice includes tasks related to understanding the illness danger signs, follow-up visits, children immunization and status on vitamin A, children nutrition, and whether health workers give a chance for caregivers to ask questions. It is likely that caregivers improve their knowledge about child care when health workers provide information and give advice to them about their children’s illnesses, treatments, and homecare management. The index on physical examination includes tasks related to assessing a child growth monitoring, malnutrition, anemia, fever, respiratory rate, chest sounds, skin turgor, and anterior fontanel (for young children). These tasks are important from a clinical point of view and they assist health workers to assess children with IMCI related illnesses. The tasks incorporated within the 4 indices are those that health workers are expected to carry out during patient-provider interaction for under-5 children in primary healthcare facilities. The inclusion of tasks in the indices, and investigating quality of care as measured by the indices was guided by the literature. Research shows significant reduction in child mortality associated with ARI case management,^19^ vitamin A administration ^10,11^, and vitamin A supplementation in young children with measles.^20,21^ Reduction of child mortality due to management of diarrheal disease,^22^ pneumonia,^23^ and severe malaria^24^ has been reported. Mortality and morbidity reduction can be expected when nutrition counseling is provided to promote breastfeeding,^25^ nutrient-rich complementary nutrition,^26,27^ and feeding of children during their illnesses.^28,29^ IMCI is the key strategy in child survival, and improving clinical quality of care related to IMCI can contribute substantially to improving child survival. Our findings support the results reported by several studies that IMCI training improves both clinical and counselling performance of health workers.^4,5,7,8,30^ Our results, however, do not support the findings reported by a study conducted in Mali.^3^ In Mali, the study found that there were several aspects of counselling activities that were not positively affected by the IMCI training intervention.^3^

In this study, we measured observed quality of care in the context of the IMCI training program. Measuring quality of care by observing how health workers perform during their interaction with patients may have the drawback of Hawthorne effect.^31,32^ In the context of our study, this means that the way health workers take history of patients, share information with caregivers, provide counseling/medical advice to caregivers, and conduct physical examination of children may be superior when they are being observed by surveyor(s) than their usual practice. It is assumed that Hawthorne effect may have occurred among health workers in treatment group and control group at similar extent. From this, it implies that treatment effects may not have been affected by the Hawthorne effect. Apart from the Hawthorne effect, our data collection approach has the advantage of objectively assessing various aspects of clinical quality of care through direct observation. It is argued that measuring clinical quality of care through direct observation has several advantages over other methods of data collection.^33^

There are some limitations in our study. First, health workers were not selected randomly from primary healthcare facilities to attend the IMCI training courses, and the purposeful selection of health workers may have biased the IMCI treatment effects to some extent which could be either underestimation or overestimation of the training effects on quality of care. Second, possible recall bias on the part of some health workers to correctly remember whether they had received IMCI training in the last 12 months or beyond is another issue. Misclassification of IMCI training status with respect to the threshold of 12 months may have occurred. Third, during data collection we did not distinguish between formal IMCI training courses (eg, conducted by the MoPH) and informal IMCI training activities (eg, conducted by the NGO who manages the respective health facilities). Therefore, some health workers may have categorized any IMCI training activities conducted at health facilities into the category of IMCI training in the last 12 months. Furthermore, health workers were not provided with any definition of what we meant by IMCI training. So it is possible that health workers responded to the question on training status as long as they recalled receiving or not receiving IMCI training from a source. Some health workers may have perceived on-the-job IMCI training as having received training in the past. Fourth, the tasks included in the indices are not inclusive of all the tasks required to be carried out by health workers for managing children with IMCI related illnesses. This is because either such information was not collected (eg, administering tablets of zinc for children with diarrhea) or the collected information could have biased the quality score if included in the index. For example, prescribing medicine for sick children was not included in the quality score because all sick children may not need prescriptions. The question was asked for all children, regardless of whether the child being examined needed to be given a prescription. We did not include data collected on 6 tasks to minimize bias in the treatment effects. However, data from all other tasks that are supposed to be carried out by health workers at primary healthcare facilities were included in the indices.

Further research is recommended to study the effects of informal training such as supervision and on-the-job IMCI training for health workers on clinical quality of care. The current model of formal IMCI training courses needs to be studied for sustainability and cost effectiveness. Alternative models to ensure more decentralized ways of conducting IMCI training courses and activities should be explored and assessed. Another venue to examine is the association between IMCI training and correct assessment, classification, and treatment of young children by health workers. Other aspects of IMCI relate to community involvement (including community health workers), patient outcomes, patient satisfaction, and patient health seeking behavior. These aspects need to be studied in future research.

## Conclusion

IMCI training courses, conducted on regular bases for health workers can improve quality of care for under-5 children in primary healthcare facilities in Afghanistan. It is recommended that IMCI training programs where applicable need to be studied to identify more cost effective and sustainable ways of implementing them within the healthcare systems of developing countries.

## Ethical issues

The data for the study come from the Afghanistan National Health Facility Assessment for which the Institutional Review Board of the Afghan Ministry of Public Health (MoPH), Kabul, Afghanistan provided ethical approval.

## Competing interests

Authors declare that they have no competing interests.

## Authors’ contributions

ET contributed to the concept/ study design, data analysis and interpretation, drafting and finalizing the manuscript. SASA contributed to the drafting of the manuscript, and administrative and technical support. KN contributed to the supervision of data collection, obtaining funding, administrative and technical support, and critical revision of the manuscript.

## Authors’ affiliations

^1^Department of Epidemiology and Biostatistics, School of Population Health, The University of Auckland, Auckland, New Zealand. ^2^Child and Adolescent Health Department, Afghan Ministry of Public Health, Kabul, Afghanistan. ^3^Silk Route Training and Research Organization (SRTRO), Kabul, Afghanistan.

## Key messages

**Implications for policy makers**

- Integrated management of childhood illness (IMCI) training courses conducted for health workers at primary healthcare facilities are effective in improving the process measures of clinical quality, and such courses should be provided to them regularly.
- Should the findings from our study be used in the design and implementation of IMCI training programs, a major limitation of our study needs to be considered that health workers from public healthcare facilities were not randomly selected into the treatment group and control group. Selection of health workers for IMCI training courses was made by their managers based on the health workers’ training needs and the requirements of IMCI algorithm in Afghanistan. This means replication of IMCI training programs from our study to other settings should be gradual, and the program implementation should be monitored and evaluated before expanding it to subnational or national levels.

**Implications for public**

Quality of care as measured by observing various tasks carried out by health workers during patient-provider interaction is the focus of this study. This study provides recommendations for policy-makers to focus meticulously on integrated management of childhood illness (IMCI) training for health workers who would learn more on how to improve communication with caregivers of children, and on how to comply with clinical procedures for assessing unwell children, conducting physical examination of children and providing counseling and medical advice for children’s caregivers.
